# Supplementary material for: High resolution cryo-EM and crystallographic snapshots of the actinobacterial two-in-one 2-oxoglutarate dehydrogenase
Source: Nat Commun. 2023 Aug 10;14:4851. doi: 10.1038/s41467-023-40253-6 (PMC10415282; doi:10.1038/s41467-023-40253-6)
Supplement: Supplementary file 3 — Description of Additional Supplementary Files [file 41467_2023_40253_MOESM3_ESM.pdf]

Description of Additional Supplementary Files

Supplementary Movie 1: Representative 3D-view and 360° rotation along two perpendicular axes of the 2.26 Å resolution EM map for OdhA in complex with succinyl-phosphonate. Map sections colored corresponding to protein chains in the final molecular model (one color per chain).

Supplementary Movie 2: Representative 3D-view and 360° rotation along two perpendicular axes of the 2.29 Å resolution final EM map for the OdhA-OdhI complex. Map sections colored corresponding to protein chains in the final molecular model (one color per chain).

Supplementary Movie 3: Representative view of twisting and tilting movements of the OdhA E1o domain inferred from the 3D variability analysis of single particle EM data. The movie shows the twisting of the E1o domains around the hexamer plane, as well as tilting movements of the longitudinal axes of the E1o domains, which deviate from their average positions on the three-fold axis of the hexamer. Calculations made by cryoSPARC v. 3.2 on the OdhA-succinyl phosphonate complex dataset; movie assembled through UCSF ChimeraX v.1.3.
