# Supplementary material for: High resolution cryo-EM and crystallographic snapshots of the actinobacterial two-in-one 2-oxoglutarate dehydrogenase
Source: Nat Commun. 2023 Aug 10;14:4851. doi: 10.1038/s41467-023-40253-6 (PMC10415282; doi:10.1038/s41467-023-40253-6)
Supplement: Supplementary file 7 — Reporting Summary [file 41467_2023_40253_MOESM7_ESM.pdf]

nature portfolio

Corresponding author(s): Bellinzoni, Marco

Last updated by author(s): Jul 14, 2023

Reporting Summary

Nature Portfolio wishes to improve the reproducibility of the work that we publish. This form provides structure for consistency and transparency in reporting. For further information on Nature Portfolio policies, see our Editorial Policies and the Editorial Policy Checklist.

Statistics

For all statistical analyses, confirm that the following items are present in the figure legend, table legend, main text, or Methods section.

n/a	Confirmed	
☐	☒	The exact sample size (*n*) for each experimental group/condition, given as a discrete number and unit of measurement
☐	☒	A statement on whether measurements were taken from distinct samples or whether the same sample was measured repeatedly
☒	☐	The statistical test(s) used AND whether they are one- or two-sided<br>*Only common tests should be described solely by name; describe more complex techniques in the Methods section.*
☒	☐	A description of all covariates tested
☒	☐	A description of any assumptions or corrections, such as tests of normality and adjustment for multiple comparisons
☐	☒	A full description of the statistical parameters including central tendency (e.g. means) or other basic estimates (e.g. regression coefficient) AND variation (e.g. standard deviation) or associated estimates of uncertainty (e.g. confidence intervals)
☒	☐	For null hypothesis testing, the test statistic (e.g. *F*, *t*, *r*) with confidence intervals, effect sizes, degrees of freedom and *P* value noted<br>*Give P values as exact values whenever suitable.*
☒	☐	For Bayesian analysis, information on the choice of priors and Markov chain Monte Carlo settings
☒	☐	For hierarchical and complex designs, identification of the appropriate level for tests and full reporting of outcomes
☒	☐	Estimates of effect sizes (e.g. Cohen's *d*, Pearson's *r*), indicating how they were calculated

Our web collection on statistics for biologists contains articles on many of the points above.

Software and code

Policy information about availability of computer code

Data collection	No custom code was used for data collection. Software employed to this purpose is reported in the manuscript with the respective versions.
Data analysis	Software employed for data processing, model building, refinement or image generation in this paper include: autoPROC v1.0.5; CCP4 v8.0; CCP-EM v1.6.0; Refmac v5.8.0405; COOT v0.9.8; BUSTER v2.10.4; cryoSPARC v3.2; PHENIX v1.20-4459-000; UCSF Chimera v1.13.1; UCSF ChimeraX v1.3; PyMOL v2.5.4; SEDFIT v. 15.01; HMMER v3.3.2; mafft v7.475; ESPript v3.0. Usage of each software is reported in the Methods section.

For manuscripts utilizing custom algorithms or software that are central to the research but not yet described in published literature, software must be made available to editors and reviewers. We strongly encourage code deposition in a community repository (e.g. GitHub). See the Nature Portfolio guidelines for submitting code & software for further information.

Data

Policy information about availability of data

All manuscripts must include a data availability statement. This statement should provide the following information, where applicable:

- Accession codes, unique identifiers, or web links for publicly available datasets
- A description of any restrictions on data availability
- For clinical datasets or third party data, please ensure that the statement adheres to our policy

Atomic models described in this study, accompanied by the corresponding structure factors (for X-ray crystallographic structures) and maps (for single particle cryo-

EM) have been deposited to the Protein Data Bank (PDB) / Electron Microscopy Data Bank (EMDB), under the following accession codes: MsKGD-GarA (crystal structure), PDB 8P5R [http://doi.org/10.2210/pdb8P5R/pdb]; OdhA 97 (crystal structure), PDB 8P5S [http://doi.org/10.2210/pdb8P5S/pdb]; OdhA (cryo-EM structure with no added ligands), PDB 8P5T [http://doi.org/10.2210/pdb8P5T/pdb] / EMD-17452 [https://www.ebi.ac.uk/pdbe/entry/emdb/EMD-17452] ; OdhA-CoASH, PDB 8P5U [http://doi.org/10.2210/pdb8P5U/pdb] / EMD-17453 [https://www.ebi.ac.uk/pdbe/entry/emdb/EMD-17453]; OdhA-succinyl-CoA, PDB 8P5V [http://doi.org/10.2210/pdb8P5V/pdb] / EMD-17454 [https://www.ebi.ac.uk/pdbe/entry/emdb/EMD-17454] (raw data available at [doi.org/10.15151/ESRF-ES-514136397]); OdhA-SP, PDB 8P5W [http://doi.org/10.2210/pdb8P5W/pdb] / EMD-17455 [https://www.ebi.ac.uk/pdbe/entry/emdb/EMD-17455]; OdhA-OdhI complex, PDB 8P5X [http://doi.org/10.2210/pdb8P5X/pdb] / EMD-17456 [https://www.ebi.ac.uk/pdbe/entry/emdb/EMD-17456]. Previously published structural models cited in the paper include the PDB entries 1EAB [http://doi.org/10.2210/pdb1EAB/pdb], 2XTA [http://doi.org/10.2210/pdb2XTA/pdb], 2XT6 [http://doi.org/10.2210/pdb2XT6/pdb], 4QCJ [http://doi.org/10.2210/pdb4QCJ/pdb], 6I2Q [http://doi.org/10.2210/pdb6I2Q/pdb], 6R29 [http://doi.org/10.2210/pdb6R29/pdb], 6ZZI [http://doi.org/10.2210/pdb6ZZI/pdb]. Source data are provided as a source data file, containing raw data relative to enzymatic activity measurements, analytical ultracentrifugation (Supplementary Fig. 2), SPR experiments (Supplementary Fig. 12b/c) and OdhA size-exclusion chromatography profile (Supplementary Fig. 20).

Human research participants

Policy information about studies involving human research participants and Sex and Gender in Research.

Reporting on sex and gender	No human research involved in this study.
Population characteristics	Not applicable
Recruitment	Not applicable
Ethics oversight	Not applicable

Note that full information on the approval of the study protocol must also be provided in the manuscript.

Field-specific reporting

Please select the one below that is the best fit for your research. If you are not sure, read the appropriate sections before making your selection.

☒ Life sciences ☐ Behavioural & social sciences ☐ Ecological, evolutionary & environmental sciences

For a reference copy of the document with all sections, see nature.com/documents/nr-reporting-summary-flat.pdf

Life sciences study design

All studies must disclose on these points even when the disclosure is negative.

Sample size	Nature and size of samples for X-ray diffraction and cryo-EM data processing are described either in the Material and Methods section, Tables 1 and 2, and/or mentioned in the main text when appropriate. Binding studies were performed with a series of 8 ligand concentrations (Supplementary Figure 12), enzymatic assays were performed on a set of 24 data points (decarboxylase activity assays via DCPIP reduction) or 60 data points (ODH activity measurements via NADH detection).
Data exclusions	Collected data and any filtering process (e.g., filters applied to images, particles or classes in EM) are described in the Methods section.
Replication	All biochemical/biophysical experiments have been thoroughly replicated. Representative experiments were chosen among a set of at least three replicates. Figure legends state this information where applicable.
Randomization	Data randomization not applicable to x-ray crystallography or cryo-EM data analysis. For biophysical experiments, experimental parameters (e.g., protein and substrates concentrations, buffers) must be chosen carefully with reference to available data, and possibly optimized, in order to to allow exploitable accurate data collection.
Blinding	Blinding, in its usual meaning as concealing information to a group of individuals/participants in order to reduce possible bias (e.g. in a clinical study), is not relevant for this paper that deals with structural and biochemical data. The nature of samples and chosen groups (including possible data exclusions) must be known.

Reporting for specific materials, systems and methods

We require information from authors about some types of materials, experimental systems and methods used in many studies. Here, indicate whether each material, system or method listed is relevant to your study. If you are not sure if a list item applies to your research, read the appropriate section before selecting a response.

Materials & experimental systems

n/a	Involved in the study
☒	☐ Antibodies
☒	☐ Eukaryotic cell lines
☒	☐ Palaeontology and archaeology
☒	☐ Animals and other organisms
☒	☐ Clinical data
☒	☐ Dual use research of concern

Methods

n/a	Involved in the study
☒	☐ ChIP-seq
☒	☐ Flow cytometry
☒	☐ MRI-based neuroimaging
